# Broadband Eddy Current Measurement of the Sheet Resistance of GaN Semiconductors

**DOI:** 10.3390/s24051629

**Published:** 2024-03-01

**Authors:** Ghania Belkacem, Florent Loete, Tanguy Phulpin

**Affiliations:** 1 ESME Research Lab, 38 rue Molière, 94200 Ivry-sur-Seine, France; ghania.belkacem@esme.fr; 2 Laboratoire de Génie Électrique et Électronique de Paris, CNRS, Centrale Supélec, Université Paris-Saclay, 91190 Gif-sur-Yvette, France; tanguy.phulpin@centralesupelec.fr

**Keywords:** eddy-currents, non-destructive measurements, epitaxy, GaN, coil probe, electrical conductivity, sheet resistance

## Abstract

Although the classical four-point probe method usually provides adequate results, it is in many cases inappropriate for the measurement of thin sheet resistance, especially in the case of a buried conductive layer or if the surface contacts are oxidized/degraded. The surface concentration of dislocation defects in GaN samples is known to challenge this kind of measurement. For the GaN sample presented in this study, it even totally impaired the ability of this method to even provide results without a prior deposition of gold metallic contact pads. In this paper, we demonstrate the benefits of using a new broadband multifrequency noncontact eddy current method to accurately measure the sheet resistance of a complicated-to-measure epitaxy-grown GaN-doped sample. The benefits of the eddy current method compared to the traditional four-point method are demonstrated. The multilayer-doped GaN sample is perfectly evaluated, which will allow further development applications in this field. The point spread function of the probe used for this noncontact method was also evaluated using a 3D finite element model using CST-Studio Suite simulation software 2020 and experimental measurements.

## 1. Introduction

The knowledge of the electrical conductivity of semiconductor materials has become a critical factor in the development of devices in the field of photovoltaic or power electronics applications. The need has, over the last decade, deeply increased with the demand for energetically efficient power components for computing [1], sustainable [2] or grid applications [3]. Gallium Nitride (GaN) has remarkable intrinsic properties, with a high thermal conductivity and a wide bandgap (3.4 eV), allowing it to have a high breakdown voltage, a high electron saturation speed and an increased energy density [4]. It is consequently a semiconductor of choice when it comes to designing more efficient electronic structures such as field effect or bipolar transistors [5]. Their reduced power loss, reaching up to 80% [6], emphasizes their economic interest for a large variety of applications. In both the scientific and industrial fields, great care is also paid to the purity and homogeneity of those materials before and during the manufacturing of a device [7].

The sheet resistance of the materials of interest is usually easily measured by a classical four-point probe measurement, but in some cases, this method fails to provide a measurement of the sample. If the contacts are too poor due to surface oxidation or inhomogeneous conductive layers caused by surface defects such as dislocations, or if the conductive layer of interest is buried under an insulating one, the current source will fail to apply the current needed to measure the contact.

The four-point probe measurement also considers that the resistivity is characterized as if the material thickness were semi-infinite. Experimental setups [8] showed that if the tip distance, multiplied by factor 5, is smaller than the material thickness, then the resistance can be derived without correction factors or considering the material thickness. When dealing with thin sheets of conductive material, correction factors depending on the form factor of the sample must be taken into account, which can cause accuracy questions [9].

Consequently, over the last decades, the fast, contactless and non-destructive characterization of samples using eddy current sensing methods has proven to be a valuable tool [10,11,12]. Among the differences, one must note that the eddy currents penetrating into a material are frequency-dependent and that the four-point probe measures along a specific line, whereas the eddy current setup is able to average the measure over a specific area. Recently, a new patented method [13] was introduced that enhanced the existing eddy current devices by allowing them to characterize the sample over a wide frequency range (GHz), thus enabling them to characterize a wide variety of materials and an extended sheet resistance range compared to existing commercial devices. In [14], the method was successfully applied to the measurement of silicon wafers and validated by correlating the results to four-point probe measurements. Its efficiency was also demonstrated on ITO nanolayers deposited on AsGa in [15].

In this study, we were interested in the electrical conductivity measurement of an epitaxially grown and doped GaN sample that was impossible to measure when first measured with a four-point probe method on the raw material. In a second time, after an ionic cleaning and the deposition of gold pads, the sheet resistance of the sample was finally estimated at 0.5 Ω · cm. We demonstrate the benefits of our multifrequency eddy current setup for the characterization of samples with poor contact resistance, buried conductive layers, or even to samples on which one does not wish to deposit metallic contact pads since the induction of the 2D eddy current in the conductive layer is not affected by the surface state of the sample. Considering the reduced size of our sample due to the fabrication process, FDTD simulations are also presented in order to evaluate the effective probed surface of such a method and ensure that no side effects would jeopardize the results.

## 2. Experimental Setup

### 2.1. Contactless Multifrequency Eddy Current System (MFEC)

**Figure 1:**
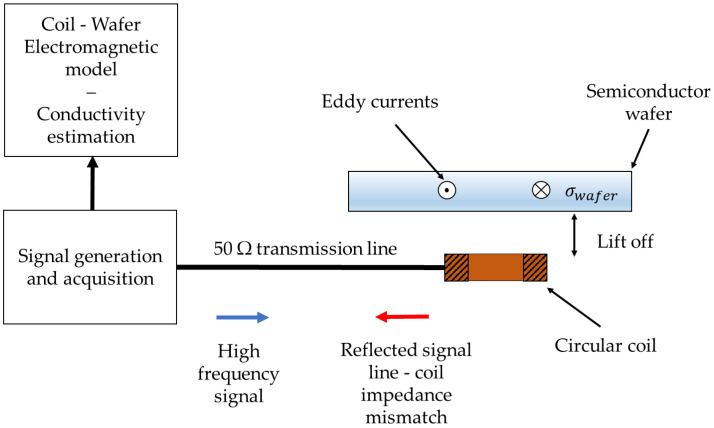
Experimental multi frequency eddy current (MFEC) setup. A multicarrier signal is sent and reflected at the coil-line impedance mismatch.

In this section, the multifrequency eddy current experimental setup introduced in [13,14] and used for the characterization of our GaN sample is described. This setup injects a broadband signal into a transmission line terminated by a probing coil. The coil, excited by an alternating signal, induces eddy currents within the conductive or semiconductor layer, leading to the generation of an induced magnetic field and a modification of the total magnetic field inside and around the layer (Figure 1). Consequently, the coil impedance changes according to the properties of the layer, such as its conductivity and thickness. The magnetic field is not affected by the presence of an insulating layer, which can be regarded as air (${µ}_{r}=1$). Therefore, the eddy current method can be successfully used to induce eddy currents in a conductive layer underneath an insulating one without being affected by the latter.

This original setup measures the reflected signal resulting from the impedance mismatch between the transmission line and the coil. Consequently, the impedances of the coil placed in the air or perturbed by a sample placed in its vicinity can be calculated according to Equation (1):

(1) $$Z_{wafer/air}\left(\omega \right)=Z_{c}\frac{1+\rho_{wafer/air}\left(\omega \right)}{1-\rho_{wafer/air}\left(\omega \right)}\;Z_{wafer/air}=R_{wafer/air}+iX_{wafer/air}$$

where $Z_{wafer/air}\;$ and $\rho_{wafer/air}$ are, respectively, the impedance and reflection coefficients of the coil in air alone or perturbated by a sample. $Z_{c}$ is the characteristic impedance of the transmission line.

The sensing coil used in this study was designed as a single-turn printed planar coil on a FR4 PCB board, with an outer radius of $r_{1}=$ 2.8 mm, an inner radius of $r_{2}=$ 2.3 mm, and a copper thickness of 35 µm.

In [16], Dodds and Deeds have described a model in which they showed that the normalized impedance variation $\frac{\delta Z}{X_{air}}\;$ due to the presence of the semiconductor wafer with respect to a reference measurement in air can be expressed as a function of the angular frequency ω, wafer conductivity, wafer thickness, and lift-off. The lift-off is defined as the distance between the exciting probe and the sample under inspection. The insulating layer thickness (when present) has to be taken into account in the conductivity estimation process as if it was air. The major challenges when measuring the conductivity of the material concern a balance between the optimum investigation frequency, lift-off and the geometry of the probe. As a matter of fact, the model shows that to a given frequency, the device will be sensitive for only a given range of sample thickness and conductivity. Consequently, the test signal injected down the transmission line, covering a [1 MHz–1 GHz] range, allows for efficient estimation of the conductivity of the sample over an extended sheet square resistance range and sample thickness range.

### 2.2. Finite Element Model and Simulation

**Figure 2:**
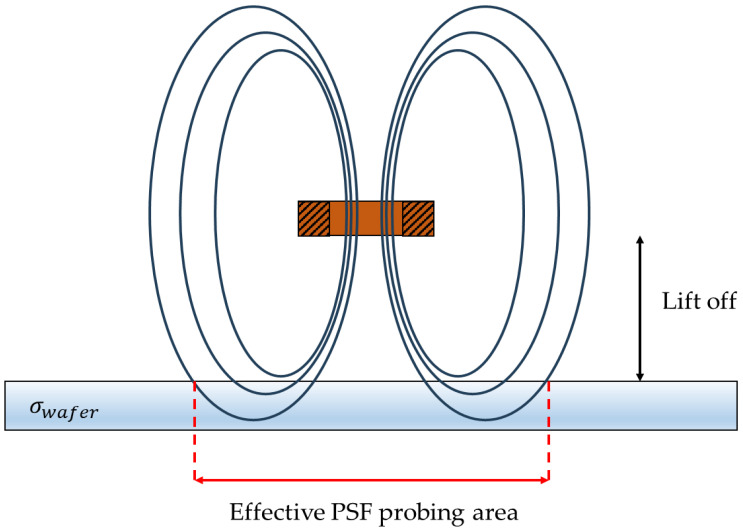
Schematic of the spatial distribution of the magnetic field over of a circular coil eddy current probe. The effective probing area of the sensor is directly related to the point spread function of the sensor inducing eddy currents in the sample under test.

In this section, we were interested in determining the effective probing area of the kind of planar coil used since it was not previously evaluated and is an important factor to consider when dealing with small samples. This parameter is an important parameter. The point spread function (PSF), which can be directly related to the spatial distribution of the magnetic field generated by the coil [17] is an important parameter to consider when designing and interpreting the conductivity of the sample measured. As shown in Figure 2, the magnetic field seen by the sample strongly depends on the lift-off. Consequently, the value measured would be an image of the conductivity of the probed area where the eddy currents are induced. The probed area corresponds to the section of the PSF by a cutting plane at a lift-off distance from the coil. Quite logically, considering the spatial extension of the field, one would expect the PSF to be wider than the radius of the coil itself [17,18] and to decrease in magnitude as a function of the lift-off. It is thus important to estimate its width compared to our coil to ensure that the effective probing section is not larger than the sample itself. Otherwise, the analytical electromagnetic model used for fitting the conductivity may not be valid since it considers an infinite layer of conducting material. If the magnetic field overlaps with the edges of the sample, the accuracy of the estimation would be affected [19].

Some analytical solutions for magnetic field mapping, which involve the derivation of the potential vector, have been described in the literature [16] for particular configurations, but, in the general case, it is often necessary and easier to rely on commercially available software such as Computer Simulation Technology (CST) Studio Suite 2020 in order to conduct a 3D FDTD analysis.

**Figure 3:**
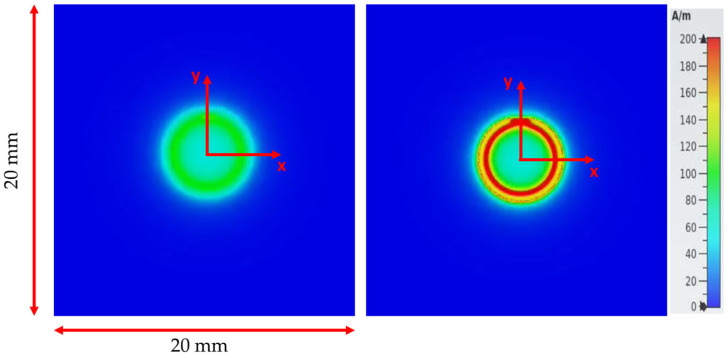
A 2D map of the magnetic the field magnitude, in air alone, for a (**left**) 400 µm and (**right**) 70 µm lift-off at 1 Mhz.

**Figure 4:**
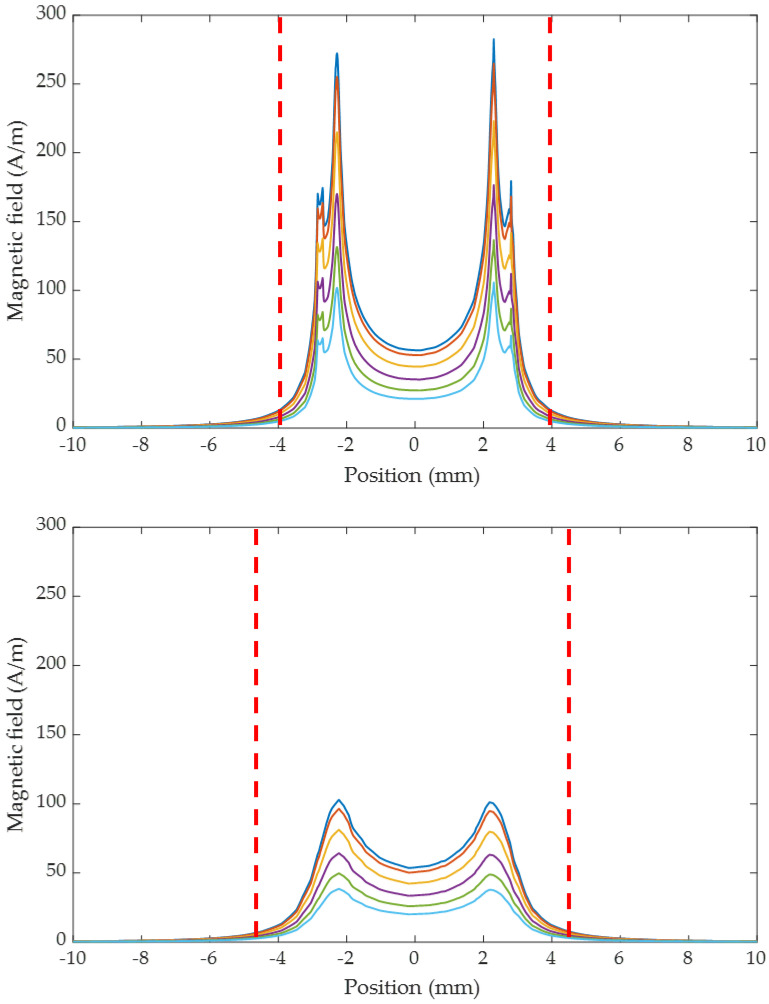
Plot of magnitude of the magnetic the field along the x direction, in air alone, for a lift-off of 70 µm (**top**) and 400 µm (**bottom**) over a 1 MHz–1 GHz range: (**-**) 1 MHz, (**-**), 200 MHz, (**-**) 400 MHz, (**-**) 600 MHz, (**-**) 800 MHz, (**-**) 1 GHz. (**- -**) 90% field limit.

In this section, the coil probe structure presented in Figure 1 was modeled and simulated over the 1 MHz–1 GHz range in the air alone or with a sample for two lift-off values of 70 µm and 400 µm. The 2D map of the magnitude of the magnetic field computed over the coil in air alone is shown in Figure 3 for two lift-offs. Very interestingly, the associated cut plots presented in Figure 4 for several frequencies show that the PSF has a very specific “M-shaped” structure caused by the air core and the small copper track width to coil radius ratio. As the frequency increases, the field is more closely localized at the surface of the coil, and the field tends to be more homogeneous. In Figure 4, for a 400 µm lift-off, the field at the coil center is about 50% lower than the one above the copper track, while for a 70 µm lift-off, the field is decreased by almost 90% and therefore mainly located above the copper track itself.

In order to quantify the spatial extension of the field (probing area), we empirically chose to evaluate the radius, which includes 90% of the field. This 90% field limit, illustrated by the dashed lines on Figure 4, is constant over the frequency range for a given lift-off: 3.9 mm and 4.5 mm for lift-offs of, respectively, 70 µm and 400 µm. Those radiuses are, respectively, 39% and 60% larger than the external radius of the coil. Although the probing area is independent of frequency. However, the field strength is dramatically affected and reduced as a function of increasing frequency and lift-off. 

Those results emphasize the importance of properly characterizing the PSF of the eddy current sensors for assessing the sample area measured and interpreting the sheet resistance estimated.

## 3. Experimental Result on the Measurement of a GaN Sample

GaN samples often contain surface dislocation due to the strongly different lattice constants and thermal expansion coefficients, as well as defects formed by lattice mismatch and residual impurities (C, O or N) [20]. The GaN layers are therefore challenging to measure with a classical four-point probe because of their low contact quality without depositing metallic contact pads.

**Figure 5:**
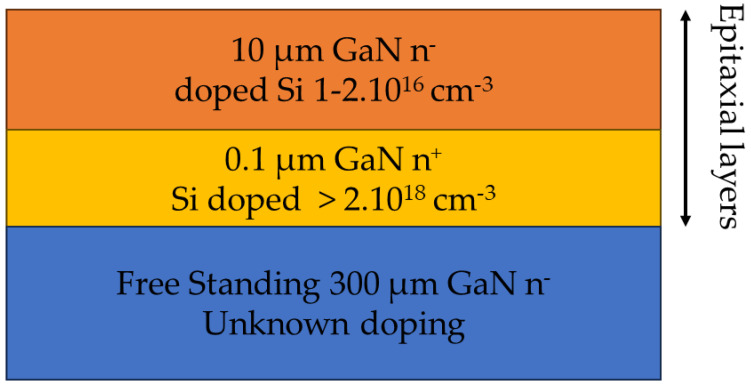
Structures and doping of the GaN sample layers deposited by epitaxy.

The sample we were willing to characterize and present in Figure 5 consists of three stacked layers of different doping concentrations. The top and inner layers of, respectively, 10 µm and 0.1 µm were grown by epitaxy on the free-standing layer of 300 µm. Their respective doping concentration were 2.10^16^ cm^−3^, 2.10^18^ cm^−3^ and unknown. The sample side was a 12 mm × 12 mm square.

At first, despite several attempts, the sample could not be measured on both sides using a four-point probe hall measurement on the raw material at ambient temperature with a 302 Lucas Labs four-point probe setup. In a second time, after an ionic cleaning and the deposition of gold pads, the sheet resistance of the sample was finally estimated at 0.5 Ω.cm and the mobility was estimated at 170 cm^2^V^−1^s^−1^. One explanation could be the presence of intrinsic structural defects such as Ga-O or Ga-OH [21] or CO or CH [22] at the interface, or the oxidation of the surface with ${Ga}_{2}0_{3}$ [23,24] leading to a degradation of the SRH recombination as well as the adsorption of carbon or hydrogen impurities.

The eddy current method proposed in Section 2 is therefore a relevant solution for thin film measurements since the induced eddy current induced in the whole volume of the sample by the magnetic field is purely 2D [22] and does not depend on the surface conductivity. Furthermore, the resistivity can be determined even though the thickness of the specimen is greater than the penetration depth of the induced currents. The sample was characterized over the 1 MHz–1 GHz range with 1 MHz steps, and the conductivity was estimated using an electromagnetic model based on [13].

The sample was measured with a 400 µm lift-off, and the sheet resistance of the sample was estimated to be 1.28 Ω per square. This value is in the same range as the one with the hall measurement; nonetheless, both measurements were made at room temperature but at different times and places. It was demonstrated that the carrier mobility of GAN and consequently the sheet resistance [25] exhibit strong dependence on the temperature [26,27] explaining the gap between those measurements. Knowing the carrier concentration, the electron mobility could be deduced since the conductivity is proportional to the product of mobility and carrier concentration. One must note that the conditions studied in Section 2 are satisfied, preventing some edge effect from impairing the measurement. Satisfyingly, the MFEC is able to measure samples efficiently and easily without worrying about the contact quality or having to deposit contact pads. Even if the conductive layer was buried inside two isolating layers, we would still be able to measure it very clearly.

## 4. Conclusions

The development of efficient resistivity imaging capabilities is a must-have for the characterization process of semiconductors along the production chain. This paper shows the benefits of applying our patented broadband eddy current measurement method to the characterization of GaN, which was difficult to measure with a classical four-point probe hall method without having to realize the deposition of metallic contact pads. After recalling the measurement principle, it was shown how the method could satisfactorily measure the square resistance of a small, three-layer GaN sample grown and doped by epitaxy. Three-dimensional FEM simulations were realized in order to correctly estimate the probing section of the sensing coil utilized in order to ensure that no edge effects were impairing the measurement. The spatial extension of the point spread function of our eddy current sensors was characterized with respect to the radius of the coil. Finally, it was shown how the setup managed to precisely estimate its sheet resistance.

## Data Availability

No new data were created or analyzed in this study. Data sharing is not applicable to this article.
